# Typhoid Fever

**Published:** 1872-03

**Authors:** 


// HALL'S

JOURNAL OF HEALTH.

VOL. XIX.—MARCH, 1872.—NO. III.

### TYPHOID FEVER

Means typhus-like, that is, similar to typhus fever; and typhus means stupor, that being a palpable characteristic of the fever; the body is oppressed with disease, and the mental faculties are weighed down — no animation, no liveliness; and as the malady progresses there is no notice taken of anything; there seems to be a stupefied condition of the whole man, with incoherent mutterings. It is accurate enough for all practical purposes for the people, to say that typhoid is an exaggerated form of typhus; that both should be called by the same name of

EXCREMENTAL FEVER, for then the common people would at once know the nature of the disease, would at once seek out its cause, remove it, and thus promptly abate the malady, not only in the individual, but in the neighborhood where prevalent. Typhoid fever, typhus fever, ship fever, jail fever, camp fever, are one and the same disease; for the cause of them all is one and the same — breathing into the lungs, swallowing into the stomach, human excrement; that is, what has been thrown from the body, or out of the body: from the body includes the perspiration, odors, fumes, coming from the skin and lungs; out of the body means the urine, and more particularly that which passes in a solid form from the bowels. The things which are breathed into the lungs and swallowed into the stomach find their way into the blood, poison it, make it thicker, arrest its flow, cause it to clog up in the small blood-vessels, derange the circulation, and finally arrest the working of the whole machinery of the body, and the man is dead.

Typhoid fever can be caused in an hour, and death may ensue in the next twenty-four, according to the concentration of the matters in the atmosphere, and the time of exposure to them.

In the editor's book on "Sleep," it is narrated that John Howard, of immortal memory, found that there were dungeons in Cornwall, England, only a hundred years ago, where several prisoners had to sleep; there was a room seven feet by five, in which there were living three persons chained together. Their provision was passed down to them through a hole in the floor, and the foulness of the air coming up through that hole, into the faces of the jailer and his wife, was so noisome, that both of them became ill and died in a few days. There were other dungeons about seven feet each way, with only one inlet for air, a hole over the door four inches broad and eight long; they contained three prisoners each, who were confined there every night in winter, lasting from fourteen to sixteen hours. In the "Chink" of the Plymouth jail, there was a "diabolical dungeon," eight feet wide, five and a half high, and seventeen long, the only air-hole seven inches by five. In this pen three men had been confined for two months, with all that passed from their bodies during that time. When Howard visited it, the door had not been opened in five weeks.

There were prisons of this kind all over England, in the very days when Pope and Johnson lived, and others of a great name. The result was that the jail fever, our typhus and typhoid fever, raged throughout England, slaying its tens of thousands, spreading its terrible contagions in such a deadly manner, that prison physicians refused to engage their services for those who had jail fever.

These examples are given to show the terrible effects resulting from the mere breathing of human emanations. Drinking them in water bubbling from the spring at the foot of the hill, although it seems as clear as crystal, poisons the blood quite as effectually.

On one occasion, in a New York village, typhoid fever prevailed to an alarming extent. It was observed, however, that it attacked only those families which were supplied with water from a certain hydrant; on a very minute investigation it was ascertained that a drain pipe from a privy leaked into the pipe which conveyed the water to the hydrant; the pipes were repaired, and the fever at once disappeared.

Not long ago, and not far from New York city, there was a splendid country-seat on an elevated position, commanding one of the most beautiful outlooks in America; it had the reputation of unusual healthfulness. There were eight persons in the family; one after another was taken ill with typhoid fever; out of the eight, six died. There had not been a case of sickness in the house for twelve years. Investigations set on foot by the family physician showed the following facts: A few weeks before the disease appeared, the pump in the well failed, and a servant brought water from a spring at the foot of the hill. At length the spring failed in consequence of the drought; resort was then had to a little running stream near by, for several weeks. This brook was supplied with water from little streams higher up, which ran through several farm yards, receiving of course the drainage from the surface, bringing into them the contents of kitchen-sinks, privies, etc. The sickness came on with nausea first, then looseness of bowels, and as the disease gained intensity, the water continuing to be used, the blood became more impure, more poisonous, at length developing malignant typhoid fever. Recently

TYPHOID FEVER

made its appearance in the upper part of New York city, in a German settlement. The physician was satisfied that there must be, somewhere near, a source of excremental poison. There was a running spring at the bottom of the hill, pure, sparkling, and bright, while the taste of it was unexceptionable; the owner of the spring was loud in his praise of its waters. A quantity of this water was placed in a bottle, and after allowing it to stand several hours, a sediment was seen at the bottom which, on examination, proved to be as purely excrement as if it had been taken direct from a privy.

It is known that Prince Albert, the husband of Queen Victoria, died of typhoid fever. From his exalted position, no one supposed that he could have been exposed to the causes of such a fever, since he could command all possible aids to cleanliness of human living. But on investigation it was found that his death resulted from breathing, habitually, the atmosphere of a privy, thus: at Windsor Castle he had a library in one of the most desirable apartments of the building; this library was his favorite place of resort, and in it he spent a large portion of his time. As it was known that typhoid fever was the result of breathing or drinking filth, it was resolved to ferret out its locality. It was eventually ascertained that before the castle was built there was an old drain which passed under its site; in digging for it, its direction was found, and tracing it along with great minuteness, it was discovered that immediately under the library there was a break in the sewer, which allowed the foul gases to escape constantly, day and night, into the library above.

The civilized world has very recently had its attention absorbingly directed to the illness of the present Prince of Wales, son of Prince Albert. Typhoid fever carried him to the very verge of the grave, from which, however, he s owly recovered. The interests of science as well as humanity demanded an investigation of the causes of the malady in this instance. The prince lived about a hundred and twenty miles from London, in a retired part of the country, amid the hills, not far from the sea-coast. In coming to his house, day after day, from visits and hunting excursions, he had to pass some yards where manure was prepared from human offal. He was generally attended by two other persons, one a young nobleman, a direct descendant of the celebrated Lord Chesterfield, and the prince's body servant. The latter died, as also the nobleman, during the illness of the prince.

Another nobleman having had occasion to pass these yards but twice, was within twenty-four hours attacked with a serious illness, the symptoms being like those which present themselves in the forming stages of typhoid fever. The party were more liable to attacks of sickness from so casual an exposure from two distinct causes, which are here mentioned in consequence of their every-day practical bearing.

First, their modes of life were not such as to give a strong constitution and that vigor of circulation which is able to repel disease. Second, returning home late in the evening or night in a state of fatigue, from the hunts of the day or other recreations, and very likely hungry also, their debilitated systems were unusually open to the baneful influence of bad airs and poisonous emanations. In addition, later investigations show that the prince was subjected to identically the same causes which resulted in his noble and lamented father's death — breathing the atmosphere of privy drains. The tide closing the mouths of these drains first, and then running up into them, drove the gases into the houses in some cases with violence sufficient to blow out a candle. The house of the prince was one of them.

THE NATIONAL HOTEL DISEASE is within the easy memory of the reader. Very many of the inmates were taken suddenly ill; indeed, from its suddenness and prevalence men did not hesitate to say that it was the result of a conspiracy to poison the incoming President of the United States, who had taken rooms at that establishment. Some died in a short time; others lingered for weeks and slowly recovered; some went abroad for their health, and died in foreign lands; some were attacked who had taken but a single meal there, and had not slept in the building at all. An extended official investigation took place, witnesses being examined on oath. In the evidence it appeared that the drains of the city had been dammed up by a freshet, in such a way that all the sewers of the hotel were full, as also those connected with the privies, with the result that on entering those on the lower floor, the filthy matter from beneath spirted up between the joints of the flooring at the first foot-fall upon it.

This same damming up of privies and sewers is constantly occurring in multitudes of places in Great Britain, at unusually high tides, with the result that typhoid fever is becoming more and more common in that country every year, and also more and more fatal, averaging about twenty thousand deaths every twelve months from typhoid fever alone. From the statements made it appears that

1st. If a case or two of typhoid fever occurs in any family, the cause should at once be sought for in the air breathed, or in the water used for drinking and cooking.

2d. It is safest to use water which comes from a spring, or well, or fountain which is higher, in a more elevated locality, than any human habitation, at least within a mile or two; and safer still, if above all barn-yards, chicken-roosts, pig-pens, and privies.

3d. Human excrement should be carried away from the dwelling without one moment's unnecessary delay.

4th. In cities, water-closets should be kept most scrupulously clean, and should be so constructed, and located, that a window should open from them immediately out of doors; and any architect who makes a different arrangement should be considered a disgrace to his profession.

5th. No gentleman's country-seat should have more than one water-closet in the building, and that to be used only in case of severe sickness, and after bed-time.

6th. Privies in the country should be at least fifty yards from the dwelling and far below the spring or water supply.

7th. The privy should have no vault or pit, but should be on the level ground, with dry earth as convenient as paper, to be used by each one, as a matter of intelligent principle and conscience, to cover what is passed. Who shall not say that the instinct of the domestic cat has not been implanted to teach men a sanitary law; the lesson only learned at the expiration of six thousand years!

8th. Once a week let the whole amount, earth and all, be removed to some suitable place for a manure heap; the surface to be quickly covered with ashes or dry earth, to absorb any odor left, and to be ready for the next deposit.

9th. Every drain of every house which receives what is deposited in water-closets and "kitchen-sinks," should be self-ventilating outside of the walls of the house for every second of the twenty-four hours.

